# Targeting fibroblast growth factor (FGF)-21: a promising strategy for metabolic dysfunction-associated steatotic liver disease treatment

**DOI:** 10.3389/fphar.2025.1510322

**Published:** 2025-04-22

**Authors:** Xinyue Cui, Quanhao Sun, Haiqiang Wang

**Affiliations:** ^1^ First Clinical School of Medicine, Heilongjiang University of Chinese Medicine, Harbin, China; ^2^ Department of Gastroenterology, First Affiliated Hospital, Heilongjiang University of Chinese Medicine, Harbin, China

**Keywords:** fibroblast growth factor 21, metabolic dysfunction-associated steatitic liver disease, metabolic dysfunction-associated steatohepatitis, liver fibrosis, FGF21 analogs

## Abstract

Metabolic dysfunction-associated steatitic liver disease (MASLD) is the predominant chronic liver disease, with its incidence increasing year by year. It has emerged as the most rapidly increasing contributor to liver-related mortality worldwide and is becoming a principal cause of end-stage liver disorders, primarily cancer of the liver and liver transplantation, hence putting a substantial economic burden on public health. The approval of Resmetirom signifies significant advancement in the treatment of metabolic dysfunction-associated steatohepatitis (MASH); nonetheless, the heterogeneity of MASLD renders it challenging for a single medication to address the requirements of all patients. Consequently, it is essential to formulate varied therapeutic approaches for distinct pathogenic causes and phases of disease. Fibroblast growth factor 21 (FGF21), a member of the fibroblast growth factor family, plays a positive and protective role in MASLD. It attenuates hepatic steatosis and lipotoxicity, ameliorates insulin resistance (IR), reduces oxidative stress, endoplasmic reticulum (ER) stress, and inflammation, as well as possesses anti-fibrotic effects. As a result, FGF21 has the potential to treat MASLD. In this review, we will address the possible mechanisms of FGF21 therapy for MASLD to facilitate the development of clinical therapies targeting FGF21 for MASLD.

## 1 Introduction

Metabolic dysfunction-associated steatotic liver disease (MASLD) is a group of chronic liver diseases in which more than 5% of hepatocytes develop steatosis in the absence of significant alcohol intake or other clear causes of hepatic steatosis and are associated with at least one cardiometabolic risk factors (CMRFs) such as overweight or obesity, insulin resistance (IR), and other metabolic abnormalities (Rinella et al., 2023). MASLD is a comprehensive term encompassing metabolic dysfunction-associated steatohepatitis (MASL) or isolated hepatic steatosis, alongside metabolic dysfunction-associated steatohepatitis (MASH), a progressive necroinflammatory form of MASLD (Huang et al., 2025). MASH characterized histologically by the presence of steatosis, lobular inflammation, and hepatocyte ballooning, with or without fibrosis (Tacke et al., 2023), potentially advancing to hepatic fibrosis, cirrhosis, and hepatocellular carcinoma (Huang et al., 2025). MASLD is the predominant cause of liver fibrosis, ultimately leading to cirrhosis (Long et al., 2023). In addition, MASLD has increasingly emerged as a significant contributor to hepatocellular carcinoma in recent years, and the incidence of MASLD-related hepatocellular carcinoma is anticipated to rise substantially in the forthcoming decade (Cannito et al., 2023). The “multiple-hit” hypothesis is currently the most universally acknowledged theory to more accurately elucidate the pathogenesis of MASLD, which includes IR, hormones released by adipose tissue, gut microbiota, nutritional factors, as well as genetic and epigenetic influences (Buzzetti et al., 2016). Resmetirom, a THRβ agonist, was recently licensed by the FDA for treating non-cirrhotic adult MASH patients with mild to advanced liver fibrosis in combination with diet and exercise (Huang et al., 2025). However, there is little information on its long-term safety and efficacy, and clinical trials with Resmetirom have concentrated on non-cirrhotic individuals, with safety and efficacy in patients with cirrhosis (especially Child-Pugh class B or C) and decompensated cirrhosis uncertain (Arvanitakis et al., 2025). Furthermore, the pathophysiology of MASLD is complex, and current medications cannot address all clinical needs. As a result, the creation of therapeutic techniques for novel targets and mechanisms remains an important area of ongoing study.

Fibroblast growth factor 21 (FGF21), a member of the fibroblast growth factor family, is synthesized mainly in the liver and adipose tissue (Falamarzi et al., 2022). FGF21 received attention as a therapeutic tool initially because it demonstrated the potential to ameliorate and correct metabolic dysfunction and reduce body weight associated with T2D and obesity (Flippo and Potthoff, 2021). It modulates lipid and glucose metabolism (Falamarzi et al., 2022) and has shown significant efficacy in improving multiple metabolic disorders, such as weight reduction, amelioration of IR, hyperglycemia, and hyperlipidemia, in intervention studies in rodents and nonhuman primates (Chen et al., 2022). FGF21 is regarded as a promising candidate molecule for MASLD treatment by targeting the signaling pathway of FGF21 with its receptors (fibroblast growth factor receptors, FGFRs) as well as the co-receptor β-Klotho (KLB), which can inhibit or reverse the process of fat accumulation, inflammatory response and fibrosis in the liver (Ritchie et al., 2020). In addition, there is a link between serum levels of FGF21 and fat content in the liver, and FGF21 may also serve as a novel diagnostic biomarker for MASLD (Rusli et al., 2016). This review intends to investigate the potential mechanisms by which FGF21 mitigates MASLD.

## 2 Molecular biology of FGF21

The mammalian fibroblast growth factor (FGF) superfamily includes 22 distinct fibroblast growth factors, categorized into seven subfamilies according to their sequence homology and functions (Dolegowska et al., 2019). FGF21 is one of the atypical members of the FGF family, possessing distinct endocrine properties (Falamarzi et al., 2022) and consists of 209 amino acids (Dolegowska et al., 2019). Unlike canonical FGFs, FGF21 has only a weak or no binding affinity for heparin, allowing it to reach the circulation and function through an endocrine mode (Zhang et al., 2006; Goetz et al., 2007). Although FGF21 mRNA expression is observable in tissues including the pancreas, muscle, and adipose (Badman et al., 2007; Hansen et al., 2015), circulating FGF21 primarily originates from the liver (Sui and Chen, 2022). FGF21 is required to bind and activate FGFRs, primarily the cell surface receptor complex consisting of FGFR1c and the co-receptor protein KLB, to act on target tissues (Spann et al., 2021). KLB enhances the binding affinity of FGFR proteins to FGF21 (Kurosu et al., 2007). FGFR1c exhibits broad expression across a range of tissues, whereas KLB is predominantly expressed in select tissues, including the liver and adipose tissue (Fon Tacer et al., 2010), thereby conferring the specificity of FGF21 signaling (Chen et al., 2022). Activation of these receptor complexes by FGF21 activates the intracellular tyrosine kinase structural domain of FGFR1c and transmits signals through phosphorylation of extracellular signal-regulated kinase (ERK) (Kharitonenkov et al., 2005; Kurosu et al., 2007; Ogawa et al., 2007; Yie et al., 2012).

## 3 FGF21 and MASLD

### 3.1 FGF21 attenuates hepatic steatosis and lipotoxicity

MASLD is characterized by hepatic steatosis (Guo et al., 2022), in which the liver accumulates FA through FA uptake from plasma and *de novo* lipogenesis (DNL) utilizing glucose-derived carbon (Esler and Cohen, 2024). FA can be oxidized by peroxisomal and mitochondrial activity in the liver (Alves-Bezerra and Cohen, 2017) and can also be secreted into the plasma as triglyceride (TG)-rich very low-density lipoprotein (VLDL) particles (Cohen and Fisher, 2013). Low steady-state hepatic TG concentrations under normal conditions are maintained when FA accumulation and elimination are in balance, whereas hepatic steatosis occurs when this balance is disturbed, resulting in the storage of excess FA in lipid droplets (Esler and Cohen, 2024). This excessive buildup of fat in the liver results in the synthesis of lipotoxic substances such as saturated fatty acids (Rada et al., 2020; Harrison et al., 2023a). Hepatic lipotoxicity occurs when persistently elevated levels of lipids and lipid metabolites are excessively deposited in the liver (Marra and Svegliati-Baroni, 2018), constituting a significant risk factor for the advancement of hepatic steatosis to MASH (Sanyal et al., 2019).

A Phase 2a clinical trial indicated that FGF21 pharmacotherapy improved hepatic steatosis in patients with MASH (Harrison et al., 2021). In rodents, exogenous FGF21 administration reduced hepatic fat content by increasing FA β-oxidation and decreasing DNL (Coskun et al., 2008).

Moreover, FGF21 or its analogs effectively decreased the expression of diet-induced adipogenic genes, including stearoyl-CoA desaturase-1 (SCD1), fatty acid synthase (FASN), and/or sterol regulatory element-binding protein 1 (SREBF1) (Keinicke et al., 2020).

Src homology three domain binding kinase 1 (SBK1) is a positive regulator of FGF21 in the liver. SBK1 phosphorylates serine 344 of the orphan nuclear receptor 4A1 (Nur77), promoting hepatic FGF21 expression while repressing the transcription of genes associated with lipid metabolism (Ahuja et al., 2023).

Subcutaneous injection of mRNA encoding human FGF21 protein achieved therapeutic levels of FGF21 and significantly reduced hepatic steatosis in diet-induced obesity (DIO) mice (Bartesaghi et al., 2022).

Lifestyle interventions, including dietary modification and exercise, are effective treatments for MASLD (Rong et al., 2022). Guo et al. (2024) found that in an *in vivo* experiment, ketogenic-diet (KD) treatment downregulated the expression of the adipogenic genes Acetyl coenzyme A carboxylase (ACC) and FASN at the transcriptional and translational levels, and upregulated the expression of the key FAO gene CPT1A, improving hepatic steatosis through the inhibition of lipogenesis and the promotion of hepatic fatty acid oxidation (FAO). KD upregulated FGF21, KLB, and FGFR1 in the liver, activating the FGF21-KLB signaling in the liver, whereas the knockdown of hepatic KLB weakened the positive effects of KD upon the amelioration of hepatic steatosis, particularly regarding the KD inhibition of lipogenesis, demonstrating the importance of hepatic FGF21-KLB signaling for KD to ameliorate hepatic steatosis. Liu et al. (2023) suggested that FGF21 induces thermogenesis through endocrine signaling to adipose tissue, hence averting adipose tissue malfunction and diminishing lipid influx into the liver. FGF21 also attenuates hepatic lipotoxicity by autocrine signaling to the liver, promoting FAO and cholesterol clearance. In conclusion, FGF21 holds therapeutic potential for MASLD by attenuating hepatic steatosis and lipotoxicity.

In MASLD patients, excessive iron accumulation in the liver correlates with disease severity and progression, and methemoglobinemia associated with iron accumulation can be observed in MASLD patients with high-fat diet (HFD)-induced MASLD models (Aigner et al., 2008; Britton et al., 2018; Kim H. Y. et al., 2020). Hepatic lipocalin-2 (LCN2) expression was markedly increased in a mouse MASLD/MASH model induced by either the non-dioxin-like (NDL) polychlorinated biphenyl (PCB) mixture Aroclor1260 or the dioxin-like (DL) PCB congener PCB126, and knockdown of LCN2 ameliorated PCB-induced lipid and iron buildup. Recombinant FGF21 was able to ameliorate hepatic steatosis and hepatic iron overload in a PCB-induced mouse model of MASLD/MASH by decreasing LCN2 expression, thereby attenuating MASLD (Kim and Yoo, 2022).

**FIGURE 1:**
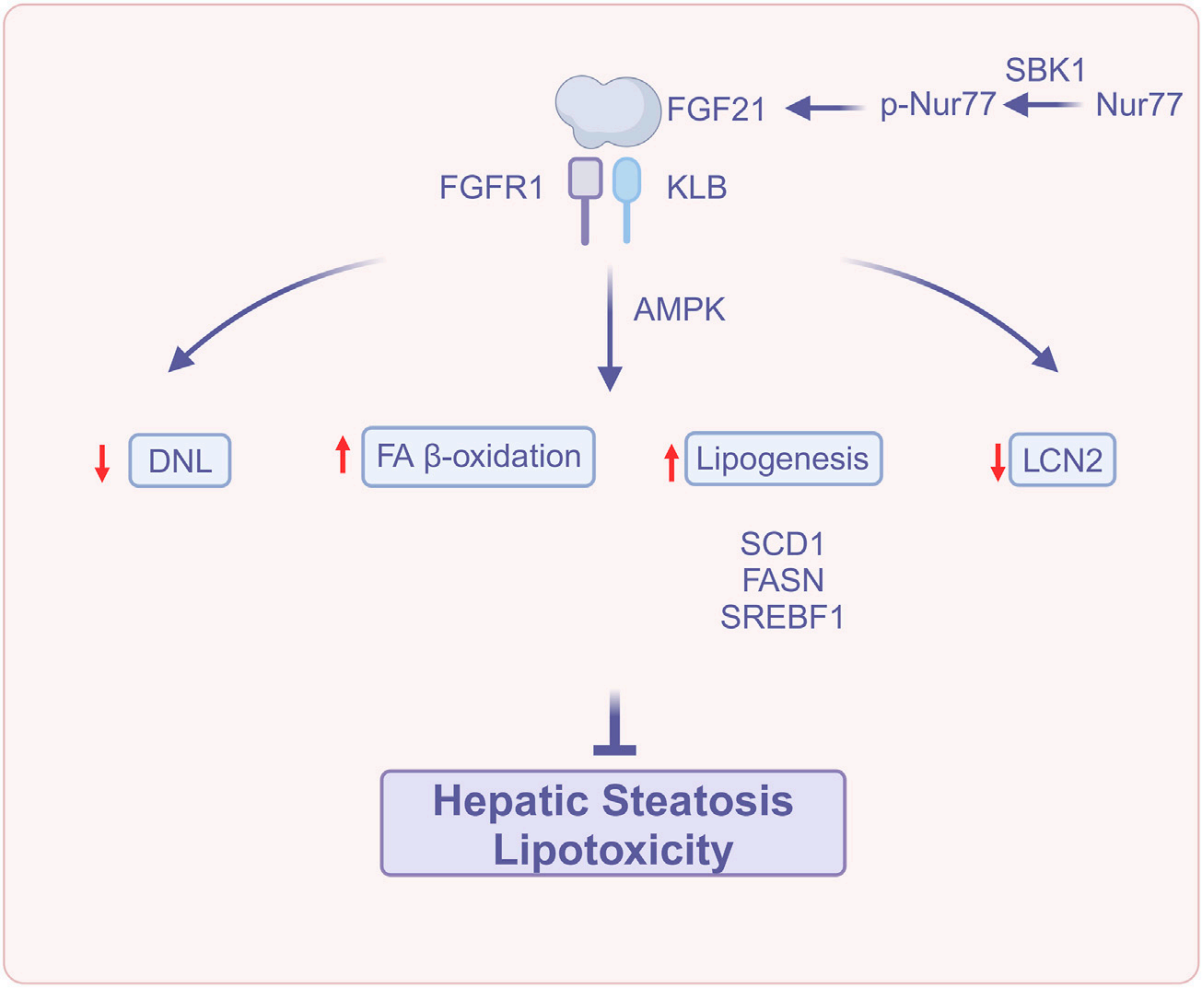
FGF21 attenuates hepatic steatosis and lipotoxicity (Created with BioRender.com). Abbreviations: DNL, *De novo* lipogenesis; FA β-oxidation, Fatty acids β-oxidation; FASN, fatty acid synthase; FGFR1, Fibroblast growth factor receptor 1; FGF21, Fibroblast growth factor 21; KLB, β-Klotho; LCN2, Lipocalin-2; Nur77, Orphan nuclear receptor 4A1; p-Nur77, Phospho-Nur77; SCD1, Stearoyl-CoA desaturase-; SBK1, Src homology three domain binding kinase 1; SREBF1, Sterol regulatory element-binding protein 1.

FGF21 reduces hepatic steatosis and lipotoxicity through a variety of mechanisms, including increased lipid oxidation, decreased lipogenesis, modulation of iron metabolism, and activation of the AMP-activated protein kinase (AMPK) signaling system (Figure 1). These outcomes suggest that FGF21 could be a target for treating MASLD.

### 3.2 FGF21 as an insulin sensitizer to improve insulin resistance

In adipose tissue, insulin inhibits lipolysis, promotes adipogenesis, regulates TG accumulation, and facilitates esterification and storage of FA (Tanase et al., 2020). FA is mostly deposited as TG in the lipid droplets of white adipose tissue (WAT) (Zechner et al., 2009). In the IR condition, the inhibition of lipolysis by insulin is weakened, leading to the catabolism of WAT and resulting in the substantial release of free fatty acid (FFA) (Haemmerle et al., 2006). The excess FFA is deposited in the liver as TG, resulting in ectopic deposits of lipids (Samuel and Shulman, 2012). Furthermore, the excessive influx of FFA induces mitochondrial dysfunction, which causes incomplete FAO and exacerbates hepatic lipid accumulation (Sekizkardes et al., 2020). In addition, IR stimulates adipogenic enzymes to promote DNL through activation of sterol regulatory element binding protein-1c (SREBP-1c) (Shimomura et al., 1999), leading to MASLD.

FGF21 can exert its acute insulin-sensitizing effect in adipose tissue by inducing uncoupling protein 1 (UCP1) expression (Gaich et al., 2013; BonDurant et al., 2017), enhancing brown adipose tissue (BAT) thermogenesis to promote glucose utilization and increase energy expenditure, inhibiting lipolysis in WAT (Arner et al., 2008), and stimulating adiponectin secretion (Turer and Scherer, 2012).

Intrahepatic ceramide accumulation can cause IR, and in insulin-sensitive tissues, adiponectin reduces ceramide accumulation (Holland et al., 2013). In addition, adiponectin also directly mediates the regulation of insulin sensitivity by FGF21 in mice (Lin et al., 2013). Consequently, FGF21 may also enhance hepatic insulin sensitivity by promoting adiponectin secretion.

Gong et al. (2016) demonstrated that FGF21 suppresses the mammalian target of rapamycin complex 1 (mTORC1) in the liver of mice, while insulin-induced mTORC1 activation and hepatic IR are exacerbated in FGF21-deficient mice; the inhibitory action of FGF21 on mTORC1 may be mediated by tuberous sclerosis complex 1 (TSC1). FGF21 has been demonstrated to function as a mTORC1 inhibitor, enhancing insulin sensitivity in a hepatocyte-autonomous manner.

Geißler et al. (2022) found that ligand-activated peroxisome proliferator-activated receptor α (PPARα) binding to peroxisome proliferator response element (PPRE) within exon one of the target gene FGF21 in mice fed HFD resulted in hypomethylation of the two CPG sites within exon one of FGF21, which led to the overexpression of FGF21 to resist IR.

The liver is the primary source of endocrine FGF21, while FGF21 originating from adipose tissue mostly exerts its effects on adipocytes in an autocrine/paracrine way (Mazuecos et al., 2021). After metabolic stress activates the c-Jun N-terminal kinase (JNK) signaling pathway, JNK inhibits PPARα-induced hepatic FGF21 gene expression and reduces circulating FGF21 to promote IR. Han et al. (2021) found that inhibition of JNK signaling in adipocytes enhances FGF21 expression and promotes autocrine/paracrine FGF21 signaling, leading to increased circulating levels of adiponectin, which may further enhance FGF21 mRNA expression and endocrine FGF21 secretion. It has been demonstrated that the autocrine/paracrine action of FGF21 in adipocytes initiates a feed-forward regulatory loop, which serves to augment hepatic endocrine FGF21 signaling.

FGF21 confers several metabolic benefits, yet its circulation concentrations are heightened in obesity and associated cardiometabolic disorders (Zhang et al., 2008). Geng et al. (2019) also demonstrated that exogenous FGF21 exhibited diminished efficacy in alleviating hyperglycemia, hyperinsulinemia, and hyperlipidemia in obese mice maintained on a chronic high-fat diet. This observation suggests the potential development of “FGF21 resistance.” Chronic high-fat diet reduces the sensitivity of FGF21 in adipose tissue, and this “resistance” can be reversed by exercise through the upregulation of PPARγ, which subsequently enhances the expression of FGFR1 and KLB genes in adipose tissue, thereby inducing the reversal of FGF21 receptor complex expression. Exercise-induced activation of PPARγ is attributed to the feedforward activation triggered by exercise between the adipose tissue FGF21 signaling cascade and PPARγ. It confirms that FGF21 signaling in adipose tissue is a crucial molecular sensor for exercise to exert metabolic benefits, such as improved systemic insulin sensitivity.

Furthermore, it is noteworthy that FGF21-mediated inter-organ crosstalk may induce hepatic steatosis through hepatic p38 activation (Liu et al., 2022). The p38 mitogen-activated protein kinase (p38MAPK) family consists of four members (p38α、p38β、p38γ and p38δ) (Canovas and Nebreda, 2021), and in MASLD, there is an increase in p38 phosphorylation (Tanida et al., 2024). MAP kinase 6 (MKK6) is the major upstream MAP2K of p38. Hepatic overexpression of MKK6 activates hepatic p38, which further increases hepatic FGF21 production by modulating X-box binding protein 1 (XBP1) and stimulates WAT lipolysis, allowing FA influx from WAT to the liver and causing hepatic ectopic lipid accumulation and IR. While FGF21 increases insulin-induced protein kinase B (Akt) phosphorylation in WAT and skeletal muscle, hence improving insulin sensitivity in peripheral tissues, p38 activation also hinders FGF21 function by promoting ubiquitination and degradation of KLB, which renders FGF21 resistant (Liu et al., 2022). In summary, FGF21 can increase insulin sensitivity and ameliorate IR, thus exerting a protective effect against MASLD.

**FIGURE 2:**
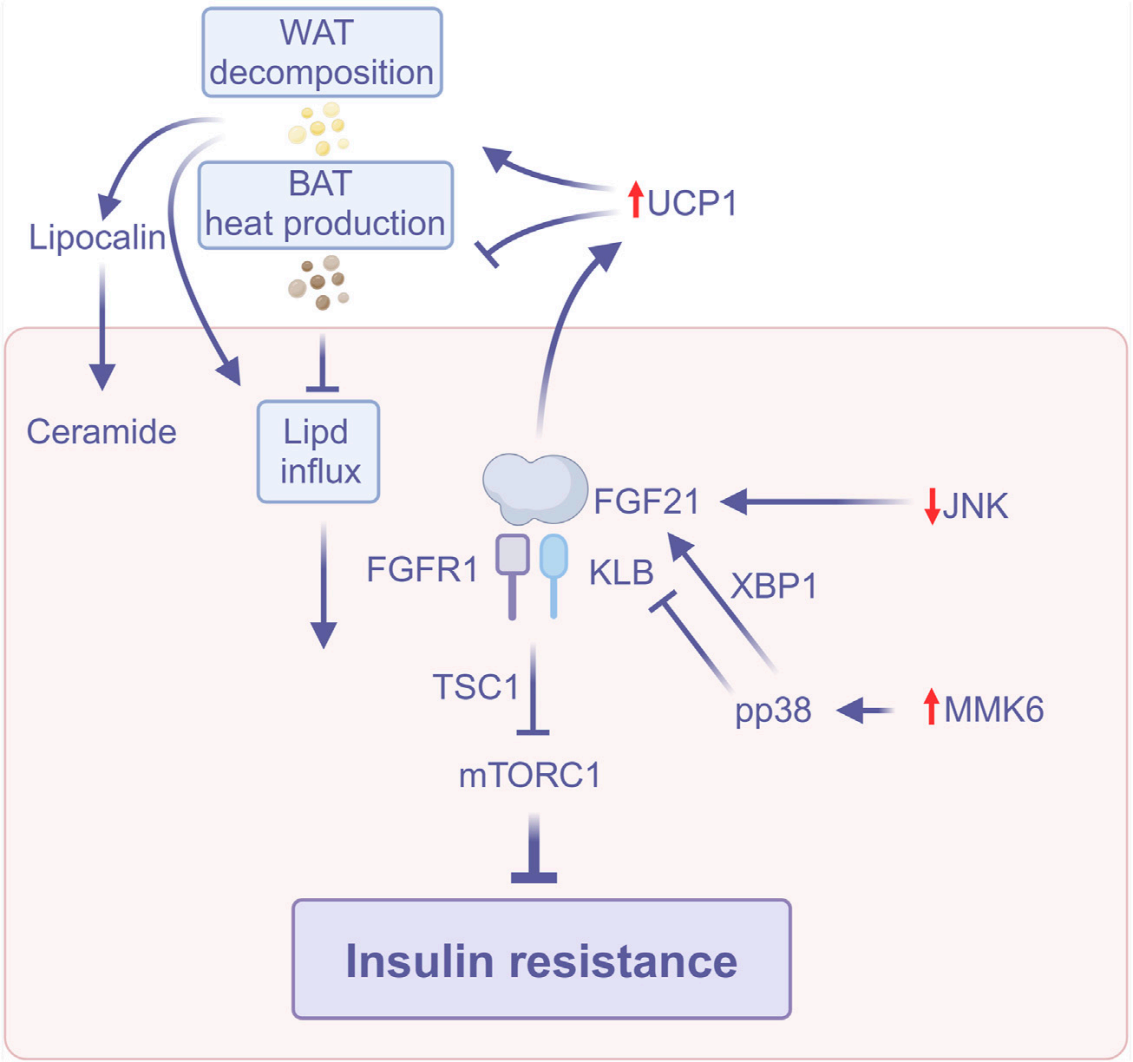
FGF21 improves insulin resistance (Created with BioRender.com). Abbreviations: BAT, Brown adipose tissue; JNK, C-Jun N-terminal kinase; mTORC1, Rapamycin complex; TSC1, Tuberous sclerosis complex 1; UCP1, Uncoupling protein 1; WAT, White adipose tissue; XBP1, X-box binding protein 1.

FGF21 improves insulin sensitivity through multiple pathways, including activation of UCP1 expression to enhance energy expenditure, inhibition of the mTORC1 signaling pathway to improve hepatic insulin sensitivity, modulation of the PPARα and JNK signaling pathways to resist insulin resistance, and reversal of FGF21 resistance through exercise. In addition, FGF21 affects metabolic homeostasis in the liver and adipose tissue by regulating inter-organ crosstalk (Figure 2). These multifaceted mechanisms of action suggest that FGF21 has important therapeutic potential in ameliorating insulin resistance and MASLD.

### 3.3 FGF21 regulates cellular stress

The endoplasmic reticulum (ER) is central to lipid metabolism, and many of the proteins that regulate lipid metabolism, such as transcription factors that regulate DNL in the liver, enzymes involved in TG synthesis, and other proteins associated with lipoprotein assembly, are located in the ER (Lee and Friedman, 2022). Excess lipid formation surpasses the ER’s ability to synthesize mature proteins, resulting in the accumulation of unfolded proteins and the activation of ER stress (Kounatidis et al., 2024). ER stress acts as a protective response to activate the unfolded protein response (UPR), enabling cells to re-establish ER quality control and reduce the buildup of unfolded or misfolded proteins in the ER, thereby restoring protein homeostasis and ER function (Zhang and Kaufman, 2004; Li et al., 2020). When ER stress lasts excessively and ER homeostasis cannot be promptly reestablished, prolonged activation of the UPR can cause inflammation, activate the inflammasome, and also activate apoptotic signaling in hepatocytes to induce their apoptosis (Hetz, 2012), thus accelerating the progression of MASLD.

Oxidative stress arises when the delicate balance between the generation of reactive oxygen species (ROS) and the antioxidant defense system’s capacity to neutralize them is disrupted (Sies, 1991). Oxidative stress is also a factor that induces liver injury in MASLD and facilitates the transition from MAFL to MASH (Polimeni et al., 2015). In hepatocytes, excessive lipid accumulation can lead to overproduction of ROS (Notarnicola et al., 2021), which further induces lipid peroxidation, promotes cytokine production and lipid accumulation, and promotes inflammation and fibrosis through multiple protein kinase and nuclear transcription factor activation pathways (Ma et al., 2021). Additionally, ROS can oxidize proteins such as antioxidant enzymes and impair their antioxidant capacity (Martín-Fernández et al., 2022), thereby exacerbating oxidative stress and creating a vicious cycle.

Self-assembled nanoparticles CH-FGF21, which are composed of chitosan and heparin (CH) utilized for the delivery and sustained release of FGF21, can increase the level of antioxidants, superoxide dismutase (SOD) activity, and glutathione (GSH) in the liver, thereby effectively mitigating the acetaminophen (APAP)-induced oxidative damage in hepatocytes (Huang et al., 2023).

Saturated fatty acids, including C16:0 (palmitic acid) and C18:0 (stearic acid), provoke ER stress, oxidative stress, inflammation, and apoptosis; their increase may result in IR and MASH (Wei et al., 2006; Fu et al., 2012; Marra and Svegliati-Baroni, 2018). Unsaturated fatty acids, like C18:1 (oleic acid), exhibit lower toxicity compared to saturated fatty acids; yet, they are rapidly assimilated by TGs and contribute to cellular lipid buildup (Listenberger et al., 2003). The administration of recombinant human serum albumin-FGF21 analog fusion protein (HSA-FGF21) restores the rise of C16:0, C18:0, and C18:1 in the livers of MASLD model mice induced by HFD-60. This indicates that the suppression of C16:0, C18:0, and C18:1 buildup by HSA-FGF21 may aid in the mitigation of liver injury and hepatic lipid buildup (Chikamatsu et al., 2023).

Furthermore, in response to ER stress, upregulated FGF21 activates multiple downstream signaling pathways, such as the AMPK/mTOR pathway, the AMPK/silent information regulator 1 (SIRT1)/peroxisome proliferator-activated receptor γ coactivator-1α (PGC-1α) pathway, *etc.*, to induce autophagy (Shen et al., 2024). Moreover, studies have confirmed that CXC chemokine receptor 3 (CXCR3), which represents autophagosome-lysosome damage and ER stress, is upregulated in MASLD mice and patients (Zhang et al., 2016), and that autophagy protects the cells from stress damage mainly by degrading damaged organelles and recycling components. Thus, FGF21 may play a therapeutic role in MASLD by enhancing autophagy (Shen et al., 2024). Taken together, FGF21 may mitigate the progression of MASLD by regulating cellular stress.

**FIGURE 3:**
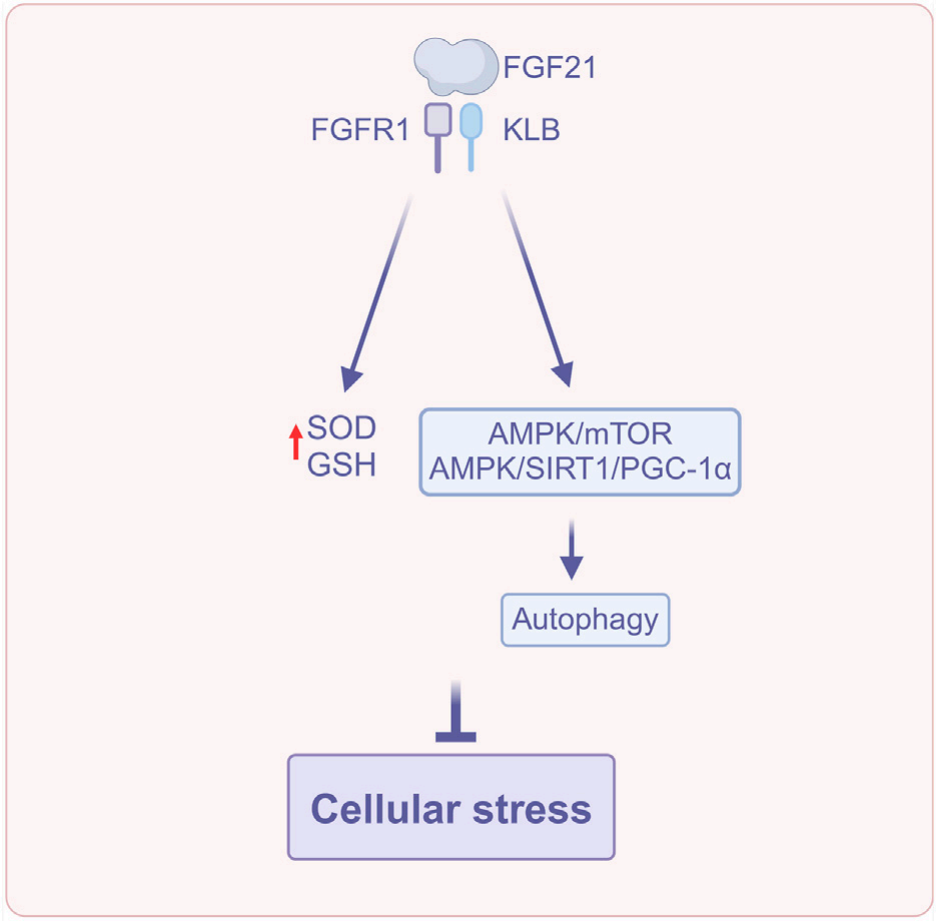
FGF21 regulates cellular stress (Created with BioRender.com). Abbreviations: AMPK, AMP-activated protein kinase; GSH, Glutathione; PGC-1α, Peroxisome proliferator-activated receptor γ coactivator-1α; SIRT1, Silent information regulator; SOD, Superoxide dismutase.

FGF21 modulates cellular stress via several routes, including reducing oxidative stress, regulating endoplasmic reticulum stress, activating the Nrf2 antioxidant pathway, correcting fatty acid metabolic imbalances, and enhancing autophagy (Figure 3). Collectively, these complex mechanisms of action contribute to the preservation of hepatic metabolic balance and the mitigation of cellular damage, thereby significantly delaying the advancement of MASLD.

### 3.4 FGF21 reduces inflammation

Hepatic inflammation is the feature that distinguishes simple steatosis from MASH. Metabolites such as lipopolysaccharide (LPS) of intestinal origin, translocated intestinal bacteria, saturated fatty acids, cholesterol, etc., to damaged hepatocytes can activate Kupffer cells (Kazankov et al., 2019). Kupffer cells, upon activation, secrete tumor necrosis factor (TNF), which further activates nuclear factor-kappaB (NF-κB) and CC chemokine ligand 2 (CCL2); they can promote the activation of pro-inflammatory macrophages and monocytes, which provide a crucial function in the initiation of hepatic inflammation (Peng et al., 2020). The inflammatory environment of the liver further activates hepatic stellate cells (HSCs), thereby fostering MASH progression (Schwabe et al., 2003). In addition, chronic ER stress can elicit a cascading response in the UPR, leading to inflammation, activating the inflammasome, and inducing hepatocyte death (Lee and Friedman, 2022). Nod-like receptor protein 3 (NLRP3), along with apoptosis-associated speck-like protein containing a CARD (ASC) and procaspase-1, forms the NLRP3 inflammasome. This complex plays a crucial role in inducing the generation of pro-inflammatory cytokines, specifically interleukin-18 (IL-18) and interleukin-1 beta (IL-1β), and initiates pyroptosis, thereby driving inflammation (Swanson et al., 2019). Chronic ER stress can also cause hepatocyte apoptosis, which in turn may activate HSCs and Kupffer cells, exacerbating hepatic inflammation (Lee and Friedman, 2022).

FGF21 reduces hepatic Kupffer cells activation, decreases monocyte infiltration, and decreases the accumulation of monocyte-derived macrophages to inhibit inflammation (Liu et al., 2023).

NLRP3 inflammasome converts the cytokine precursor pro-IL-18 to mature IL-18, and NLRP3 inflammasome is pivotal in the advancement of steatosis and liver fibrosis (Patel et al., 2015), whereas the inhibitor of nuclear factor kappa-B kinase subunit epsilon (IKBKE) is able to negatively regulate NLRP3 inflammasome (Patel et al., 2015). Oxidized low-density lipoprotein (ox-LDL) participates in inflammatory and fibrotic processes in MASH through its interaction with CD36. It is generated from low-density lipoprotein (LDL) as a reaction to oxidative stress (Houben et al., 2017). The mitochondrial uncoupling protein 2 (UCP2) diminishes ROS production (Brand and Esteves, 2005), and the ER chaperone protein heat shock 70 kDa protein 5 (HSPA5) is a pivotal regulator that mitigates the accumulation of misfolded proteins (Keinicke et al., 2020). Keinicke et al. (2020) found that FGF21 administration increased IKBKE, which inhibits the chronic nature of inflammation, and decreased the expression of IL-18 and the inflammatory biomarker lipocalin-2 (LCN2). FGF21 was shown to prevent HFD-induced inflammation. In addition, FGF21 reduces the expression of CD36 while simultaneously increasing the expression of low density lipoprotein receptor (LDLR), thereby promoting the hepatic clearance of LDL; it upregulates the expression of UCP2 and HSPA5 to mitigate oxidative and ER stress, which may also be a mechanism to inhibit hepatic inflammation.

Hepatic infiltration of IL-17A-secreting cells promotes the development of simple steatosis to MASH (Mladenić et al., 2024). Toll-like receptor 4 (TLR4)-mediated hepatocellular inflammation contributes to the development of MASLD. However, most TLR4 signaling is concentrated in nonparenchymal cells, including HSCs and Kupffer cells. Hepatic parenchymal cells constitute around two-thirds of the total liver cell population (Zheng et al., 2020), and the expression of TLR4 in hepatic parenchymal cells is very low. Zheng et al. (2020) found that FGF21 deficiency leads to upregulation of TLR4 expression in hepatic parenchymal cells, resulting in the FFA-mediated hepatic parenchymal cell-TLR4-NF-κB signaling pathway, which upregulates IL-17A expression in hepatic parenchymal cells. Recombinant human FGF21 (rhFGF21) treatment can attenuate FFA-mediated TLR4-IL-17A signaling, demonstrating that FGF21 may exert anti-inflammatory effects by inhibiting IL-17A production in the liver *via* the suppression of the hepatic parenchymal cell-TLR4-NF-κB signaling pathway. What’s more, FGF21 may also prevent the progression of MASH to HCC by inhibiting the hepatic parenchymal cell-TLR4-IL-17A axis. The above studies indicate that the anti-inflammatory effect of FGF21 constitutes a mechanism by which it inhibits the further progression of MASLD.

**FIGURE 4:**
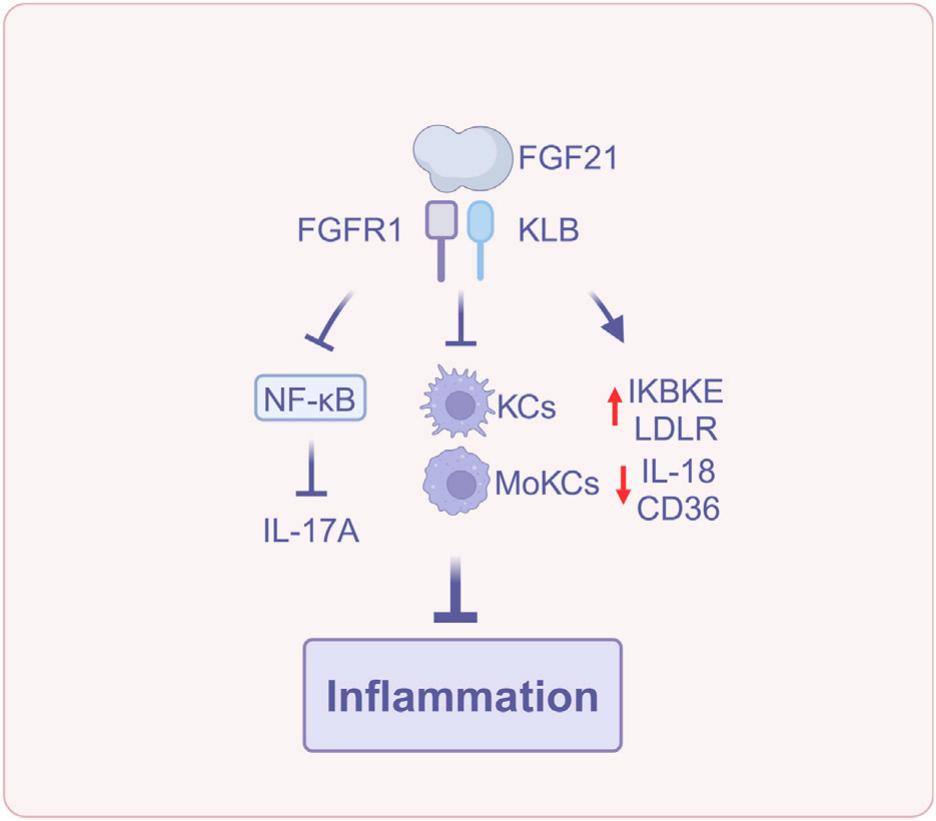
FGF21 reduces inflammation (Created with BioRender.com). Abbreviations: IL-17A, interleukin-17A; IL-18, interleukin-18; KCs, Kupffer cells; LDLR, Low density lipoprotein receptor; MoKCs, monocyte-derived Kupffer cells; NF-κB, Nuclear factor-kappa B.

The aforementioned studies indicated that FGF21 suppresses hepatic inflammation *via* various mechanisms, encompassing the regulation of Kupffer cell and monocyte activity, modulation of NLRP3 inflammasome and IL-18 expression, reduction of ox-LDL accumulation, alleviation of oxidative and endoplasmic reticulum stress, alongside the inhibition of the TLR4-NF-κB signaling pathway and IL-17A production (Figure 4). These results render FGF21 significant in suppressing inflammatory advancement in MASLD.

### 3.5 FGF21 reduces liver fibrosis

Liver fibrosis is caused by chronic liver injury, an inflammatory response, and sustained activation of fibrosis (Parola and Pinzani, 2024). HSCs are one of the major drivers of liver fibrosis in MASLD (Jain et al., 2023). Damaged hepatocytes can directly or indirectly stimulate the activation of HSCs through the release of damage-associated molecular patterns (DAMPs), while macrophages can promote the activation of HSCs by means of the release of cytokines like transforming growth factor-β (TGF-β), platelet-derived growth factor (PDGF), TNF, and other cytokines (Yan et al., 2021). Activated HSCs lose lipid-rich granules and transdifferentiate into α-smooth muscle actin (α-SMA)-positive myofibroblasts. These myofibroblasts can produce increased amounts of extracellular matrix (ECM) along with pro-inflammatory and pro-fibrotic cytokines, thereby acilitating the progression of liver fibrosis (Kamm and McCommis, 2022). In addition, activated HSCs can also induce hepatic inflammation through the secretion of cytokines (Wang et al., 2022), which further promotes the development of liver fibrosis. Fibrosis is a significant prognostic indicator of prognosis in patients with MASLD (Heyens et al., 2021). Liver fibrosis can progress to advanced cirrhosis and portal hypertension, leading to various associated complications that may ultimately result in liver failure (Parola and Pinzani, 2024).

FGF21 reduces the accumulation of lipid-associated macrophages [CD36hi resident Kupffer cells (CD36hi ResKCs), a subset of macrophages that reside in the liver and contribute to inflammation, and CD36hi monocyte-derived Kupffer cells (CD36hi MoKCs)] and scar-associated macrophages (CD9hi MoKCs), which are involved in fibrosis-related processes. This inhibits the activation of HSCs to generate collagen and, in turn, prevents the development of early liver fibrosis (Liu et al., 2023).

Beyond its effects on macrophages, FGF21 also impacts multiple signaling pathways that contribute to fibrosis. First, in a mouse model of carbon tetrachloride (CCl4) and dimethylnitrosamine (DMN)-induced liver fibrosis, FGF21 downregulated the expression levels of collagen Ⅰ and α-SMA, as well as significantly downregulated the expression of pro-fibrotic cytokine TGF-β, suggesting the TGF-β signaling pathway as a possible mechanism for its amelioration of liver fibrosis. In addition, leptin, an adipocyte-derived hormone (Cheng et al., 2020), has ERK involved in its expression, and it acts autocratically to further promote fibrosis in HSCs *via* the signal transducer and activator of transcription 3 (STAT3)/TGF-β pathway (Meng et al., 2021). Specifically, leptin activates the STAT3 signaling pathway, leading to the upregulation of TGF-β, a key pro-fibrotic cytokine. Inflammation and ROS can also induce HSCs activation (Yang et al., 2017). The suppressor of cytokine signaling 3 (SOCS3) functions as a negative regulator of leptin and mediates the anti-inflammatory mechanism by up-regulating thioredoxin *via* Nrf2 to downregulate ROS signaling (Kim G.-Y. et al., 2020). In a model of HSCs activated by PDGF-BB treatment, FGF21 downregulates several markers of fibrosis, including α-SMA, collagen I, and leptin, while also reducing the ratios of p-ERK1/2 to t-ERK1/2 and p-STAT3 to t-STAT3, as well as TGF-β expression. Additionally, FGF21 upregulates the expression of anti-inflammatory markers such as Nrf2 and SOCS3. These findings suggest that the FGF21-leptin-STAT3 axis may be a potential mechanism for ameliorating fibrosis in PDGF-BB-activated HSCs (Meng et al., 2021). Finally, leptin mediates the proliferation of HSCs through AKT phosphorylation and inhibits apoptosis of HSCs through the caspase family (Zhu et al., 2020; Jia et al., 2023). FGF21 significantly decreases the ratio of p-AKT to t-AKT, downregulates the B Cell lymphoma-2 (Bcl-2) gene, and upregulates bcl-2-associated X protein (Bax) and caspase-3. This reduces HSC proliferation and increases apoptosis, which may contribute to the resolution of liver fibrosis (Meng et al., 2021).

PsTag-FGF21 is a long-acting FGF21 analog, a fusion protein with the long-acting tag PsTag fused to human FGF21, which has superior pharmacokinetics and pharmacology (Ji et al., 2024). Ji et al. (2024) reported that PsTag-FGF21 improves hepatic fibrosis, alanine aminotransferase (ALT) and aspartate aminotransferase (AST) levels, and collagen content in 2 mouse models of MASH-associated fibrosis. The first model utilized the Gubra amylin NASH (GAN) diet, while the second employed a Western diet containing 21.1% fat, 41% sucrose, and 1.25% cholesterol, along with a high-sugar drinking solution (23.1 g/L D-fructose and 18.9 g/L D-glucose), in combination with low-dose CCl4 to promote fibrosis development. Additionally, it decreases gene expression of collagenⅠ, actin alpha 2 (ACTA2), and TGF-β1, which are closely related to the activation of HSCs, inhibitor of matrix metallo proteinases (TIMP1), and matrix metalloproteinase-8 (MMP-8) relating to the accumulation of ECM; it also increased gene expression of MMP2, MMP9 and MMP13, which are associated with the degradation of ECM, suggesting that it has a therapeutic effect on MASH-associated fibrosis. Mechanistically, PsTag-FGF21 regulates the conversion of macrophages from a pro-inflammatory and pro-fibrotic phenotype characterized by high expression of lymphocyte antigen 6C (Ly6C^hi^) to an anti-inflammatory and anti-fibrotic phenotype marked by low expression of Ly6C (Ly6C^lo^). This conversion occurs *via* the upregulation of the orphan nuclear receptor nuclear receptor subfamily four group A member 1 (NR4A1), and NR4A1 regulates insulin-like growth factor-1 (IGF-1) expression through N-terminal intrinsically disordered region (IDR) transcription, thereby inhibiting HSCs activation. The aforementioned studies suggest that FGF21 can participate in the regulation of MASLD fibrosis through multiple mechanisms.

**FIGURE 5:**
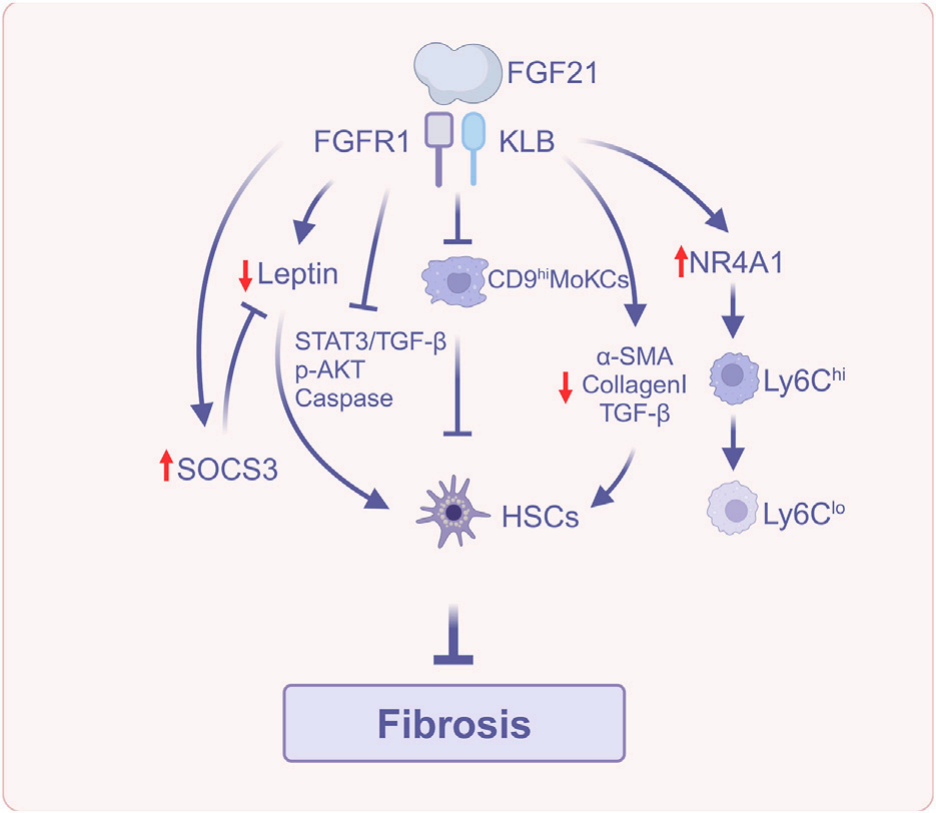
FGF21 reduces liver fibrosis (Created with BioRender.com). Abbreviations: α-SMA, α-smooth muscle actin; AKT, Protein kinase B; HSCs, Hepatic stellate cells; NR4A1, Nuclear receptor subfamily four group A member 1; Ly6C, Lymphocyte antigen 6C; SOCS3, Suppressor of cytokine signaling 3; STAT3, Signal transducer and activator of transcription 3; TGF-β, Transforming growth factor-β.

In summary, FGF21 exerts its anti-fibrotic effects by regulating macrophage polarization, modulating key cytokine signaling pathways, and directly influencing the activity of HSCs (Figure 5). The several antifibrotic mechanisms collaborate to enable FGF21 to successfully impede the advancement of MASLD-related liver fibrosis.

## 4 Clinical study of FGF21 analogs for the treatment of MASLD

The beneficial effects of FGF21 on lipid metabolism, insulin resistance, oxidative stress, inflammation, and hepatic fibrosis make this molecule a promising candidate for the treatment of MASLD. Preclinical studies and experimental research suggest that FGF21 may significantly alleviate the pathological processes involved in the progression of MASLD, with the potential to inhibit the transition from MASL or isolated hepatic steatosis to MASH and even cirrhosis. However, from preclinical evidence and promising effects observed in laboratory studies to the implementation of effective and safe therapies on a larger scale, much remains to be done, and conclusive evidence from clinical trials is still lacking (Chen, 2023; Negroiu et al., 2024).

Native endogenous FGF21 has poor pharmacokinetic (PK) properties, with a short half-life of less than 2 h, making it unsuitable for clinical application (Geng et al., 2020). In contrast, FGF21 analogs have a longer half-life and greater *in vitro* stability than natural FGF21 (Zhao et al., 2024). Therefore, the development of FGF21 analogs holds significant importance for the treatment of MASLD.

### 4.1 Pegozafermin

Pegozafermin (BIO89-100) is a glycopolyethylene glycolized FGF21 analog (Rosenstock et al., 2023). A Phase 1b/2a clinical trial has shown that weekly subcutaneous injections of Pegozafermin 27 mg for 20 weeks could significantly improve liver histology in patients with MASH and fibrosis. In addition, there was a notable improvement in serum aminotransferases, noninvasive fibrosis tests, lipids, glycemic control, and body weight compared to baseline, and it was well tolerated (Alkhouri et al., 2023). In another Phase 2b, multicenter, double-blind, randomized, placebo-controlled trial, pegozafermin administered subcutaneously at 30 mg weekly or 44 mg biweekly for 24 weeks in patients with MASH and stage F2 or F3 (moderate or severe) fibrosis could improve fibrosis and reduce liver fat. Fibrosis markers such as iron-corrected T1 and the Enhanced Liver Fibrosis (ELF) test scores, liver stiffness, FibroScan-AST (FAST) scores, type III collagen amino-terminal pre-peptide (Pro-C3) levels, and Fibrosis 4 Score were reduced, as was the volume of the liver and spleen (Loomba et al., 2023). A Phase I single ascending dose study in healthy subjects found that subcutaneous abdominal injections of pegozafermin at single dose levels of 9.1 mg or higher resulted in up to a 51% reduction in serum TG, up to a 36% increase in high-density lipoprotein cholesterol (HDL-C), and up to a 37% decrease in low-density lipoprotein cholesterol (LDL-C). Notably, the most significant increase in serum adiponectin levels was observed on Day 8 of dosing and persisted through Day 29. In the most clinically significant dosage range of 3–39 mg, the pharmacokinetic half-life is 55–100 h (Rosenstock et al., 2023).

### 4.2 Efimosfermin Alfa

Efimosfermin Alfa (formerly BOS-580 or LLF580) is a novel genetically engineered variant of human FGF21, which improves the thermodynamic stability of the FGF21 fragment by introducing a disulfide bond, reduces its protein hydrolytic stability, and is fused with the human IgG (IgG1 subclass) Fc structural domain at its N-terminus (Rader et al., 2022). In a multicenter, double-blind, parallel-controlled trial, three doses of Efimosfermin Alfa 300 mg administered subcutaneously every 4 weeks to obese, mildly hypertriglyceridemic patients resulted in a 54% reduction in serum TG, a 7% decrease in total cholesterol (TC), a 12% decline in LDL-C, a 36% increase in HDL-C, and a 52% reduction in hepatic fat over a 12-week period. Liver function tests, including ALT, AST, alkaline phosphatase (ALP), liver fibrosis score, and Pro-C3, also showed improvement. In addition, Efimosfermin Alfa decreased insulin and c-peptide levels and IR and increased adiponectin levels. Overall, Efimosfermin Alfa is generally safe and well-tolerated (Rader et al., 2022).

### 4.3 Efruxifermin

Efruxifermin, also known as AKR-001 or AMG876, is a long-acting Fc-FGF21 analog (Kaufman et al., 2020; Puengel and Tacke, 2023). Unlike other analogs containing a single FGF21 fragment, efruxifermin is a bivalent analog composed of two covalently linked bivalent analogs of FGF21 chains, thus enhancing affinity of the receptor and reducing dissociation. A multicenter, randomized, double-blind, placebo-controlled Phase 2b trial assessed the impact of 24 weeks of efruxifermin at doses of 28 mg or 50 mg on liver histology in individuals with MASH and stage F2 or F3 fibrosis. In the liver biopsy analysis set (LBAS), 39% of participants in the efruxifermin 28 mg group and 41% in the 50 mg group exhibited improvement in at least one stage of fibrosis without any deterioration of MASH. In addition, efruxifermin reduced markers of liver injury within 4 weeks, with a very high percentage of ALT and AST levels returning to normal by the 24th week (Harrison et al., 2023b). In another double-blind, controlled, Phase 2b study, it was observed that in patients with T2D and MASH combined with fibrosis stages 1–3 (F1-F3) who were stably administered with glucagon-like peptide one receptor agonist (GLP-1RA), after administration of Efruxifermin 50 mg once weekly for 12 weeks, the patients had a 65% reduction in hepatic fat fraction (HFF) and an improvement in noninvasive markers of liver injury, fibrosis, glucose, and lipid metabolism, and the combination with GLP-1RA was found to be safe and well tolerated (Harrison et al., 2024).

### 4.4 Zalfermin

Compared to natural FGF21, Zalfermin (NNC0194-0499, 15) has superior chemical and metabolic stability, natural FGFR selectivity, reduced immunogenicity risk, and favorable biophysical and formulation characteristics that facilitate prolonged refrigerated storage and ambient application, enabling subcutaneous administration. Phase 1 clinical trials assessing safety, pharmacokinetics, and efficacy (NCT03015207, NCT04722653, and NCT03479892) endorse the continued development of zalfermin, which is presently undergoing a Phase 2b clinical trial for the treatment of MASH (NCT05016882) (Sass-Ørum et al., 2024).

## 5 Clinical study of FGF21-receptor agonistic antibodies for the treatment of MASLD

A different strategy for FGF21 drug development involves the selective targeting of elements within the FGFR1c/KLB co-receptor complex (either FGFR1c and KLB together or KLB alone), which may yield the metabolic effects of FGF21 necessary for MASH treatment while circumventing the possible adverse effects linked to the extensive activation of the FGF21 signaling pathway. The bispecific monoclonal antibody (mAb) simultaneously binds KLB and FGFR1c, leading to the development of an active FGFR1c/KLB complex (Raji et al., 2025).

### 5.1 BFKB8488A

BFKB8488A is a bispecific antibody that targets FGFR1c and KLB. A Phase 1b randomized, blinded, placebo-controlled multiple dose-escalation study indicated that BFKB8488A was well tolerated in patients with T2DM or MASLD, resulting in decreased triglycerides, enhanced HDL levels, and a trend towards improved liver health markers in both cohorts. In patients with MASLD, liver fat content was significantly diminished in the BFKB8488A-treated group, with reductions of 13.0%, 34.5%, and 49.0% in the low, medium, and high exposure groups, respectively, compared to a mere 0.1% in the placebo group (Wong et al., 2023).

### 5.2 MK-3655

MK-3655 (formerly NGM313), a humanized monoclonal antibody that targets KLB and stimulates the FGFR1c/KLB complex, is utilized in pre-cirrhotic MASH patients. A phase 2b, randomized, multicenter, double-blind, placebo-controlled trial revealed that patients with pre-cirrhotic MASH (NAS ≥4 and MASHCRN Fibrosis Score 2 or 3) were assigned in a 1:1:1:1 ratio to receive either MK-3655 (50 mg, 100 mg, 300 mg) or placebo subcutaneously every 4 weeks for a duration of 52 weeks. In the interim analysis (IA), the relative reduction in hepatic fat content from baseline compared to placebo was deemed inadequate to warrant the continuation of the experiment. At 24 weeks, the percentage decrease in hepatic fat content from baseline was 19.1%, 19.0%, and 26.1% for the MK-3655 50 mg, 100 mg, and 300 mg groups, respectively, compared to 11.0% in the placebo group. At 52 weeks, the proportion of individuals achieving MASH remission without fibrosis deterioration (16.7%, 14.3%, and 17.6%) and those exhibiting ≥1 stage of fibrosis improvement without steatohepatitis exacerbation (22.2%, 38.1%, and 29.4%) was greater in the MK-3655 cohort compared to the placebo cohort (5.9% and 17.6%); however, these differences did not attain statistical significance. While MK-3655 demonstrated a slight reduction in hepatic fat and was usually well tolerated, since MK-3655 binds only KLB, on the one hand the interaction of MK-3655 and KLB may not fully activate the FGFR1/KLB co-receptor complex. On the other hand it is possible that the assembled receptor complex modifies downstream signaling. Furthermore, a more extensive activation of FGFR isoforms, including FGFR2 and FGFR3, may be necessary for good metabolic function in humans for the management of MASH (Raji et al., 2025). Consequently, the advancement of FGF21 pharmaceuticals that specifically activate the FGFR1c/KLB complex necessitates additional comprehensive research.

## 6 Clinical study of GLP-1/FGF21 dual agonist for the treatment of MASLD

### 6.1 HEC88473

HEC88473 is a novel long-acting dual agonist of GLP-1 and FGF21. The GLP-1 and FGF21 components of HEC88473 are connected through IgG4Fc, thereby prolonging the half-life to guarantee sustained pharmacodynamic efficacy. A randomized, double-blind, placebo-controlled, multiple dose-escalation Phase Ib/IIa trial indicated that patients with MASLD and T2DM receiving subcutaneous doses of 5.1 mg, 15.3 mg, 30.6 mg, 45.9 mg, and 68.0 mg weekly for 5 weeks exhibited a favorable safety profile and tolerability, with significant dose-dependent effects. Decreased hepatic fat content, elevated adiponectin levels, and markedly enhanced lipid profiles, particularly TG and LDL-C, with a peak reduction of −42.15% in TG in the 68.0 mg cohort and a peak reduction of −25.84% in LDL-C in the 45.9 mg cohort. Moreover, there was a substantial reduction in HbA1c values, along with fasting and postprandial blood glucose levels (Xiang et al., 2024). The summary of FGF21-related drugs and their corresponding clinical trials is presented in Table 1.

**TABLE 1:** Clinical trial results for FGF21-related drugs.

Brand name	Clinical trial ID	Clinical trial status	Disease	Outcome	References
FGF21 analogs					
Pegozafermin	NCT04048135	phase 1b/2a	MASH	Improvement of liver fibrosis Liver fat content↓ Improvement in liver function indicators: ALT↓,AST↓ Improvement in blood lipids: TG↓,LDL-C↓,non-HDL-C↓,HDL-C↑ Improvement in blood glucose control: HbA1c↓; body weight↓ adiponectin↑	Alkhouri et al. (2023)
	NCT04929483	phase 2b	MASH	Improvement of liver fibrosis Liver fat content↓ Improvement in liver function indicators: ALT↓ Liver volume↓ Improvement in blood lipids: TG↓; adiponectin↑	Loomba et al. (2023)
Efimosfermin Alfa	NCT03466203	phase 2	Obese; Hypertriglyceridemia	Improvement of liver fibrosis Liver fat content↓ Improvement in liver function indicators: ALT↓, AST↓, ALP↓ Improvement in blood lipids: TG↓,TC↓, LDL-C↓, HDL-C↑ Improvement in blood glucose control: Insulin↓ C-peptide↓, HOMA-IR↓; adiponectin↑	Rader et al. (2022)
Efruxifermin	NCT04767529	phase 2b	MASH	Improvement of liver fibrosis Liver fat content↓ Improvement in liver function indicators: ALT↓, AST↓, ALP↓, GGT↓ Improvement in blood lipids: TG↓, non-HDL-C↓, HDL-C↑ Improvement in blood glucose control: HbA1c↓, C-peptide↓, HOMA-IR↓; body weight↓	Harrison et al. (2023b)
	NCT05039450	phase 2b	T2DM; MASH	Improvement of liver fibrosis Liver fat content↓ Liver fat content↓ Improvement in blood lipids: TG↓, non-HDL-C↓, ApoB↓, HDL-C↑ Improvement in liver function indicators: ALT↓, AST↓ Improvement in blood glucose control: HbA1c↓; body weight↓	Harrison et al. (2024)
Zalfermin	NCT05016882	phase 2b	MASH	-	Sass-Ørum et al. (2024)
FGF21-receptor agonistic antibodies					
BFKB8488A	NCT03060538	phase 1 b	T2DM; MASLD	Improvement of liver fibrosis Liver fat content↓ Improvement in blood lipids: TG↓, HDL↑ Improvement in liver function indicators: ALT↓, AST↓; body weight↓	Wong et al. (2023)
MK-3655	NCT04583423	phase 2 b	MASH	Improvement of liver fibrosis Liver fat content↓; adiponectin↑	Raji et al. (2025)
GLP-1/FGF21 dual agonist					
HEC88473	NCT05943886	phase 1b/2a	T2DM; MASLD	Liver fat content↓ Improvement in blood lipids: TG↓, TC↓. LDL-C↓ Improvement in blood glucose control: HbA1c↓, FPG↓, PPG↓, HOMA-β↑; adiponectin↑	Xiang et al. (2024)

Abbreviations: ALP, Alkaline Phosphatase; ALT, Alanine Aminotransferase; ApoB, Apolipoprotein B; AST, Aspartate Aminotransferase; FPG, Fasting Plasma Glucose; FGF21, Fibroblast Growth Factor 21; GGT, Gamma-Glutamyl Transferase; GLP-1, Glucagon-Like Peptide-1; HbA1c, Hemoglobin A1c; HDL-C, High-Density Lipoprotein Cholesterol; HOMA-IR, Homeostatic Model Assessment for Insulin Resistance; HOMA-β, Homeostatic Model Assessment for Beta Cell Function; LDL-C, Low-Density Lipoprotein Cholesterol; MASH, Metabolic Dysfunction-Associated Steatohepatitis; MASLD, Metabolic Dysfunction-Associated Steatotic Liver Disease; PPG, Postprandial Glucose; TC, Total Cholesterol; TG, Triglycerides; T2DM, Type 2 Diabetes Mellitus.

## 7 Conclusion and outlook

Despite advancements in understanding the pathogenesis of MASLD, the “multiple-hit” theory continues to be the mainstream theory and breakthrough for its study. Nonetheless, the clinical landscape still needs to develop more effective therapeutic agents, owing to the intricate pathogenesis resulting from interactions among various organs and systems. The aforementioned preclinical and clinical studies indicate that FGF21 and its analogs show great potential in the treatment of MASLD, with significant effects in attenuating hepatic steatosis and lipotoxicity, ameliorating IR, decreasing oxidative stress, ER stress, inflammation, and antifibrosis; therefore, FGF21 may emerge as a novel therapeutic target for MASLD. However, the advancement of FGF21-based therapies encounters several challenges, including concerns regarding indications, contraindications, bioavailability, administration methods, stability, and half-life, underscoring the disparity between the success of preclinical studies and clinical implementation. Moreover, subsequent research may explore the potential of integrating FGF21 analogs with alternative therapies, including lifestyle modifications, to enhance treatment efficacy. Future research must investigate its efficacy and safety for treating MASLD in humans and perform clinical studies to enhance MASLD treatment.
